# Rating Scales for Movement Disorders With Sleep Disturbances: A Narrative Review

**DOI:** 10.3389/fneur.2018.00435

**Published:** 2018-06-07

**Authors:** Carmen Rodríguez-Blázquez, Maria João Forjaz, Monica M. Kurtis, Roberta Balestrino, Pablo Martinez-Martin

**Affiliations:** ^1^National Center of Epidemiology and CIBERNED, Institute of Health Carlos III, Madrid, Spain; ^2^National School of Public Health and REDISSEC, Institute of Health Carlos III, Madrid, Spain; ^3^Movement Disorders Unit, Neurology Department, Hospital Ruber International, Madrid, Spain; ^4^Department of Neuroscience “Rita Levi Montalcini, ” University of Turin, Turin, Italy

**Keywords:** movement disorders, sleep disorders, rating scales, Parkinson's disease, progressive supranuclear palsy

## Abstract

**Introduction:** In recent years, a wide variety of rating scales and questionnaires for movement disorders have been developed and published, making reviews on their contents, and attributes convenient for the potential users. Sleep disorders are frequently present in movement disorders, and some movement disorders are accompanied by specific sleep difficulties.

**Aim:** The aim of this study is to perform a narrative review of the most frequently used rating scales for movement disorders with sleep problems, with special attention to those recommended by the International Parkinson and Movement Disorders Society.

**Methods:** Online databases (PubMed, SCOPUS, Web of Science, Google Scholar), related references from papers and websites and personal files were searched for information on comprehensive or global rating scales which assessed sleep disturbances in the following movement disorders: akathisia, chorea, dystonia, essential tremor, myoclonus, multiple system atrophy, Parkinson's disease, progressive supranuclear palsy, and tics and Tourette syndrome. For each rating scale, its objective and characteristics, as well as a summary of its psychometric properties and recommendations of use are described.

**Results:** From 22 rating scales identified for the selected movement disorders, only 5 included specific questions on sleep problems. Movement Disorders Society-Unified Parkinson's Disease Rating scale (MDS-UPDRS), Non-Motor Symptoms Scale and Questionnaire (NMSS and NMSQuest), Scales for Outcomes in Parkinson's Disease (SCOPA)-Autonomic and Progressive Supranuclear Palsy Rating Scale (PSPRS) were the only rating scales that included items for assessing sleep disturbances.

**Conclusions:** Despite sleep problems are frequent in movement disorders, very few of the rating scales addresses these specific symptoms. This may contribute to an infra diagnosis and mistreatment of the sleep problems in patients with movement disorders.

## Introduction

A wide variety of rating scales and questionnaires for the wide range of movement disorders that can affect patients have been developed and are currently available for clinical practice and research. These instruments may be classified as “rater-based,” which are applied by a health professional or trained person, and “patient-based,” which are directly completed by patients themselves. Rater-based scales (clinician-reported outcomes measures) are used to evaluate observable signs of the disorder (e.g., tremor, rigidity, instability, myoclonus, tics) by means of clinical examination, and other non-observable aspects through interview with the patient and/or caregiver. Patient-based instruments (patient-reported outcomes measures) allow the assessment of non-observable, subjective features, and perceptions (e.g., pain, fatigue, sensations, feelings, hallucinations, health state) (1). Some effects caused by the health disorder (e.g., disability, symptoms) can be appraised by both methods.

Their simplicity of use, as well as the amount and quality of information the rating scales provide, justify why rating scales and questionnaires are widely used in clinical and research settings.

Sleep disorders are frequently present in movement disorders, as they can share pathophysiological mechanisms and damage to brain structures (2). Insomnia and sleep fragmentation are common in Parkinson's disease (PD), multiple system atrophy (MSA), progressive supranuclear palsy (PSP) (3), and different choreic disorders. REM-sleep behavior disorder appears associated to α-synucleinopathies, such as PD, Lewy body dementia, and MSA, and can be an early marker of the disease (4). Impaired sleep architecture is frequent in Tourette syndrome (5). PD and essential tremor (ET) are often associated with restless legs and nocturnal periodic limb movements (6, 7). The relevance of sleep problems in movement disorders has been acknowledged in the recent years and specific rating scales for assessing sleep have been developed and validated. However, these scales are only available for PD, and specific symptoms are not sufficiently addressed (8). Simultaneously, global rating scales for the assessment of specific movement disorders have been developed, such as the Unified Huntington's Disease Rating Scale (UHDRS), Unified Dystonia Rating Scale (UDRS), or the Unified Multiple System Atrophy Rating Scale (UMSARS), with the aim of providing a comprehensive appraisal of the clinical manifestations (motor and non-motor symptoms) of these disorders.

The aim of this study is to perform a narrative review of most frequently used rating scales for those movement disorders that comprise sleep dysfunction among their primary manifestations or sleep problems secondary to the movement disorder, with special attention to the scales recommended by the International Parkinson and Movement Disorders Society (IPMDS) Task Force (9).

## Methods

Authors made a list of the movement disorders that can course with sleep symptoms that included akathisia, chore, dystonia, essential tremor, multiple system atrophy, myoclonus, Parkinson's Disease, progressive supranuclear palsy, and tics and Tourette syndrome. The literature search, carried out using PubMed, Web of Science and Scopus, included these terms plus “sleep” and “rating scales.” In addition, the reviews published by IPMDS on these movement disorders (9) and related references from papers and personal files were examined. The IPMDS Task force classifies a scale as “recommended” if it has been used in PD, shows adequate psychometric properties, and has been used by investigators other than the original developers; as “suggested” if it has been used in PD and fulfills only one other criterion; and as “listed” if it has been used in PD but does not meet the other criteria.

The main rating scales for Parkinson's disease (PD), such as the Movement Disorders Society–Unified Parkinson's Disease Rating Scale (MDS-UPDRS) and the Non-Motor Symptoms Scale (NMSS), are also included. Table 1 lists the scales incorporated to this review. Specific instruments assessing sleep disorders in movement disorders, such as the Parkinson's Disease Sleep Scale (PDSS) and the Scales for Outcomes in PD (SCOPA)-Sleep, are reviewed in another article in this issue (8). For each rating scale, its objective, characteristics, psychometric properties, and recommendations of use are described.

**Table 1:** Rating scales for movement disorders with sleep disturbances included in this review.

**Movement disorder**	**Rating scale**	**References**	**Includes sleep assessment?**
Akathisia	Barnes Akathisia Rating Scale, BARS	(10, 11)	No
Chorea	Universidade Federal de Minas Gerais Sydenham's Chorea Rating Scale, USCRS	(12)	No
	Unified Huntington's Disease Rating Scale, UHDRS	(13)	No
Dystonia	Unified Dystonia Rating Scale, UDRS	(14)	No
	Fahn-Marsden Dystonia Rating Scale, F-M Scale	(15)	No
	Toronto Western Spasmodic Torticollis Rating Scale, TWSTRS	(16)	No
Essential tremor	Fahn-Tolosa-Marin Tremor Rating Scale	(17)	No
	Bain and Findley Clinical Tremor Rating Scale	(18)	No
	Bain and Findley Spirography Scale	(19)	No
	Washington Heights -Inwood Genetic Study of Essential Tremor Rating Scale, WHIGET	(20)	No
	Tremor Research Group Essential Tremor Rating Assessment Scale, TETRAS	(21)	No
Multiple system atrophy	Unified Multiple System Atrophy Rating Scale, UMSARS	(22)	No
Myoclonus	Unified Myoclonus Rating Scale, UMRS	(23)	No
Parkinson's Disease	Movement Disorders Society–Unified Parkinson's Disease Rating Scale, MDS-UPDRS	(24)	Yes (2 items, validated as screening tool)
	Non-Motor Symptoms Scale, NMSS	(25)	Yes (3 items)
	Non-Motor Symptoms Questionnaire, NMSQuest	(26)	Yes (5 items, validated as screening tool)
	Scales for Outcomes in Parkinson's Disease (SCOPA)-Motor	(27)	No
	Scales for Outcomes in Parkinson's Disease (SCOPA)-Autonomic	(28)	Yes (2 items)
Progressive supranuclear palsy	Progressive Supranuclear Palsy Rating Scale, PSPRS	(29)	Yes (1 item)
Tics and Tourette syndrome	Yale Global Tic Severity Scale, YGTSS	(30)	No
	Shapiro Tourette Syndrome Severity Scale, STSSS	(31)	No
	Tourette Syndrome Clinical Global Impressions scale, TS-CGI	(32)	No
	Tourette's Disorder Scale, TODS	(33)	No
	Premonitory Urge for Tic Disorders Scale, PUTS	(34)	No

## Movement disorders and rating scales

### Akathisia

Akathisia has been associated to nocturnal periodic limb movements and increased number of awakenings (35). However, the main rating scale for akathisia, the Barnes Akathisia Rating Scale (BARS) does not include sleep assessment (10). The BARS is a 4-item scale for rating the presence and severity of the drug-induced akathisia. It shows adequate reliability, validity, and responsiveness (11).

### Chorea

Results of an online survey of juvenile Huntington's disease (HD) suggests that disrupted sleep is the most prevalent common, unrecognized symptom (87%), followed by periodic limb movements, tics, and pain (36, 37). HD gene carriers complain about sleep problems, both in terms of sleep quality as well as excessive daytime sleepiness (38, 39). Sleep complaints seem to be associated with neuropathology and neuropsychiatric symptoms in HD (40). However, ratings scales for chorea or HD do not assess sleep difficulties.

The Universidade Federal de Minas Gerais Sydenham's Chorea Rating Scale (USCRS) assesses signs and symptoms of children and adults with Sydenham's Chorea and related disorders (12). It is formed by 27 items organized into three sections (behavior, activities of daily living, and motor assessment). Items are scored on a 0–4 rating scale, with higher values indicating higher severity of disability or signs. Although the scale presented a two-factor structure (motor function and ADL; behavioral) (12), a total sum score is used in most studies (41–44). Published in 2005 (12), the scale is rater-based and it is owned by the IPMDS. It has shown adequate inter-rater reliability and internal consistency (12), as well as discriminative validity by disease stage (44). The USCRS has been used in Brazil (12, 43, 44), Italy (42), and Israel (41).

The UHDRS was developed by the Huntington Study Group as a research tool, and it has been used as an outcome measure in clinical trials (13). It is formed by the following components: motor, with 15 items (45); cognitive (formed by Verbal Fluency Test; Symbol Digit Modalities Test; Stroop Interference Test), behavioral (10 items) and functional (5 items) assessments, independence scale (1 item, from 10, totally dependent, to 100, totally independent), and total functional capacity (TFC, 25 items). Internal consistency is high and the UHDRS shows satisfactory inter-rater reliability and sensitivity to change. Although it was published as annex of an article (13), it is actually owned by the Huntington Study Group and permission for use is required.

A IPMDS task force rated the UHDRS behavioral section (UHDRS-b) as a “suggested” scale for assessing severity of and screening for behavioral symptoms in patients with HD (46). The UHDRS also has a version for advanced patients (UHDRS-FAP), with satisfactory internal consistency and inter-rater reliability (47).

### Dystonia

There is a growing interest on non-motor symptoms of dystonia patients, including sleep problems (48). Sleep impairment may be a primary effect of dystonia or secondary effects of pain and medications (49). Different types of dystonia may be associated to specific sleep disorders: poor sleep quality has been described in blepharospasm, cervical dystonia patients report more daytime sleepiness than controls (50), and impaired sleep efficiency and decreased rapid eye movement (REM) sleep has been reported in blepharospasm and oromandibular dystonia (51).

On the basis of their psychometric properties and clinical and research application, a review commissioned by the IPMDS qualified five disease-specific scales as “recommended,” and two scales as “listed” for laryngeal dystonia (52). None of them assesses sleep disorders.

The UDRS (14), a rating scale for generalized dystonia, was “suggested” for use in dystonia and did not reach a “recommended” rating due to insufficient psychometric studies about responsiveness (52). The clinician assesses 14 body locations, rated for both duration (0–4 score, including half-scores) and severity (0–4 scale) of dystonia. The total score is the sum of the duration and severity ratings. Internal consistency is high, and the scale shows good inter-rater reliability and convergent validity with other dystonia rating scales (14). The UDRS is owned by the IPMDS and license for use is needed.

The Fahn-Marsden Dystonia Rating Scale (F-M Scale), a predecessor of the UDRS, was initially developed to assess primary torsion dystonia in 9 body parts (15). It has two factors, one for severity (each body part is rated from 0, no dystonia, to 4, severe dystonia) and the other for the precipitation or provoking factor (from 0, no dystonia, to 4, dystonia at rest). The scores for eyes, mouth, and neck are multiplied by 0.5 when calculating the total score, which is then obtained by summing the product of the severity, provoking, and weighting factors. The F-M Scale also includes a disability scale based on the patient's report that evaluates the impact of dystonia on seven activities of daily living. The walking item is rated on a 7-point scale and the rest of the disability items on a 0–4 point scale. The F-M scale has shown good internal consistency, inter-rater agreement, and convergent validity with other dystonia rating scales (14), as well as adequate responsiveness (52). Despite criticisms of the low contribution of some body parts to the overall score, this scale is “recommended” to assess the severity of the dystonia (52) and has been used for evaluating dystonia in many conditions and clinical trials.

The Toronto Western Spasmodic Torticollis Rating Scale (TWSTRS) (16) is a “recommended” rating scale that was designed to assess a specific condition, cervical dystonia, in clinical trials (52). It is formed by three subscales assessing motor severity (11 items), disability (6 items), and pain (3 items). The first subscale is rated by the clinician whereas the other two are patient-rated. The scoring system is not uniform and a videotape for training is available for the severity section. This scale, available in English, has been frequently used in clinical trials. Its psychometric properties are satisfactory and well-documented, including internal consistency, inter-rater agreement for the severity subscale, internal, and convergent validity, as well as responsiveness. The TWSTRS has been criticized for its complexity for clinical practice (52). Excessive daytime sleepiness in cervical dystonia was not associated with TWSTRS scores (50).

### Essential tremor

Besides motor features, essential tremor also includes non-motor symptoms such as sleep problems (53–55), which have a negative effect on quality of life (56). Excessive daytime sleepiness, shorter sleep duration (57), and restless legs complaints are more frequent in essential tremor patients than in healthy controls (8).

Despite increasing evidence of sleep problems in essential tremor, none of the currently available rating scales to assess tremor include items about sleep. The IPMDS Task Force recommends several rating scales for evaluating tremor severity.

The Fahn-Tolosa-Marin Tremor Rating Scale provides a comprehensive assessment of tremor, rated by the clinician and the patient (17). It is formed by 3 parts: tremor in 9 body parts and orthostatic tremor; action tremor in 3 tasks; and patient-reported functional disability. In addition, both clinical and patient rate a global assessment item. Items are rated on a 0–4 point scale. The scale presents good intra and inter-rater reliability (18), but there is a lack of data on other psychometric parameters (58). It has been used in many clinical trials.

The Bain and Findley Clinical Tremor Rating Scale assesses tremor severity (59) and impact on activities of daily living. For each body part (head, voice, and limbs), the clinician rates the severity of several tremor components: rest, postural, and kinetic/intention tremor. The initial 0–10 response scale was simplified to a 5-point scale with intermediate values. Inter-rater reliability was satisfactory as a whole, especially for upper limb postural and head tremor, but not for voice tremor (59). The severity scale shows good convergent validity with other tremor measures, and it was sensitive to change in clinical trials (58). With a scoring system based on subjective impression, the Bain and Findley Clinical Tremor Rating Scale is easy to apply in a diversity of conditions and circumstances, including bedside.

In the Bain and Findley Spirography Scale, action tremor is assessed through Archimedes spirals. Rating are on a 0–10 scale, and rating examples are provided (59, 19). Its reliability is good provided that raters are trained (58). This scale has been criticized for its high floor and ceiling effects; however, it shows adequate face and construct validity (58).

The Washington Heights -Inwood Genetic Study of Essential Tremor (WHIGET) Rating Scale is aimed at assessing the severity of essential tremor during the performance of several tasks (20). The clinician rates the following tremor parameters: intensity, amplitude, oscillation prevalence, and persistency of rest, kinetic and postural tremor. There are 26 items rated on a 0–3 scale. A revised version rates kinetic tremor from 0 to 4 (60). A training videotape is available (60). Test-retest and inter-rater reliability are satisfactory, as well as convergent validity with other measures of tremor (61). Some studies support the scale's sensitivity to change (62, 63). The WHIGET limits assessment to upper extremity tremor.

The Tremor Research Group Essential Tremor Rating Assessment Scale (TETRAS) is formed by two subscales (21). The performance subscale, with 9 items, rates action tremor in head, face, voice, limbs, and trunk. The activities of daily living section if formed by 12 items. Both sections use a 0–4 point scale, but performance items admit half-point scores. This short, easy to apply scale has appropriate reliability, validity, and sensitivity to change for the performance section (64–66). More psychometric studies are needed for the activities of daily living subscale.

### Multiple system atrophy (MSA)

MSA affects sleep in several ways: sleep-disordered breathing (67), sleep fragmentation, REM sleep behavior disorder, insomnia, and excessive daytime sleepiness (68). In addition, sleep study (polysomnography) are included in a list of useful tools for differential diagnosis of MSA (69). Despite this, specific rating scales for this movement disorder do not include items for assessment of sleep disturbances.

The most used scale for MSA is the UMSARS developed by the European MSA Study Group (22). It comprises four parts: Part I, historical (functional status), with 12 items; Part II, motor examination, with 14 items; Part III, autonomic examination, with items assessing blood pressure, heart rate and orthostatic symptoms; and Part IV, global disability scale. In Parts I and II, scores range from 0 to 4, and in Part IV, the scale ranges from 1 (completely independent) to 5 (totally independent). The UMSARS psychometric properties are adequate, showing high internal consistency in Parts I and II, satisfactory inter and intra-rater reliability and sensitivity to change over time (22, 70). The UMSARS can distinguish between different subtypes (parkinsonism- vs cerebellar ataxia-predominant) of MSA (71) and has been used as reference in the validation of other scales for MSA (72). It is owned and licensed by the IPMDS. A comparative review of the longitudinal performance of the UMSARS and other scales for MSA can be found in Matsushima et al. (73).

### Myoclonus

Clinical presentations of myoclonus are divided into physiological, essential, epileptic, and symptomatic (74). While physiological myoclonus can occur as jerks during sleep or sleep transitions in healthy individuals, other myoclonic sleep disorders (e.g., propriospinal myoclonus) can be identified (75) and some types of myoclonic epilepsy, characterized by abnormal sleep electroencephalogram and an activation of the paroxysms during non-REM sleep and on waking up (76).

The main tool for assessing myoclonus is the Unified Myoclonus Rating Scale (UMRS) (23). It assesses the severity and characteristics of the disorder and the associated disability. The UMRS has 73 items, grouped into five sections: patient's questionnaire (12 items, scored from 1 to 5); myoclonus at rest (8 items for frequency and amplitude, scored from 0 to 4); stimulus sensitivity (17 items, dichotomous); myoclonus with action (10 items, scored for frequency and amplitude on a 5-point scale); and functional tests (5 items, scored from 0 to 4). It also includes a global disability scale, scored from 0 (normal) to 4 (severe), and two items assessing presence (yes/no) and severity (from 0 to 3) of negative myoclonus. Components for evaluation of myoclonus-related sleep disorders are not included in this scale. The UMRS has satisfactory internal consistency and inter-rater reliability (23, 77) and is responsive to changes due to treatment (78).

### Parkinson's disease (PD)

Sleep disorders are common in PD: they can affect up to 60–90% of PD patients, with increasing prevalence as the disease progresses (7). Insomnia and sleep fragmentation, excessive daytime sleepiness, restless legs and REM-sleep behavior disorder are frequently present in PD (79). Some specific rating scales for sleep disorders in PD are currently available, but they are the object of another article in this issue. Sleep problems are included in the main multi-domain, comprehensive rating scales for PD, the MDS-Unified Parkinson's Disease Rating Scale (MDS-UPDRS) (24), and the Non-Motor Symptoms Scale (NMSS) (25).

The MDS-UPDRS is the revised version of the widely used UPDRS (80). It has four sections: Part I, non-motor experiences of daily living; Part II, motor experiences of daily living; Part III, motor examination; and Part IV, motor complications. In Part I, with 6 rater-based items and 7 self-assessed items, two questions rating nighttime sleep problems and daytime sleepiness can be used as screening tool for sleep disturbances (81). All items are scored from 0 (normal) to 4 (severe). The MDS-UPDRS has good psychometric properties and is responsive to changes due to treatment (82, 83). The minimal clinically important difference of the Part III has been calculated (84).

The NMSS, which is administered by interview, was designed to assess the burden (frequency and severity) of non-motor symptoms (NMS). It is composed of 30 items, grouped in nine domains: cardiovascular (2 items), sleep/fatigue (4 items), mood/cognition (6 items), perceptual problems/hallucinations (3 items), attention/memory (3 items), gastrointestinal tract (3 items), urinary (3 items), sexual function (2 items), and miscellaneous (4 items). In the domain sleep/fatigue, there are three questions to rate daytime sleepiness, problems falling or staying asleep and restless legs syndrome, while in the urinary domain, one item assesses nocturia. All items are scored 0–3 for severity and 1–4 for frequency. The total item score is obtained by multiplication of both aspects and domain and scale score by sum of the respective items scores. The scale, validated, and available in several languages, has good psychometric properties, including responsiveness (85–87).

An instrument related with the NMSS is the Non-Motor Symptoms Questionnaire (NMSQuest) (26), which was developed as a self-assessment of non-motor symptoms. It is composed by 30 yes/no questions, of which six address sleep disturbances: nocturia, daytime sleepiness, insomnia, vivid dreams, acting out while dreaming, and restless legs. These items have been validated and resulted useful as a screening tool for sleep difficulties in PD patients (88). The NMSQuest has good feasibility, acceptability and validity, resulting suitable for patients to flag symptoms that may be undeclared and remain untreated (89). A NMSQuest-based grading system for NMS burden has also been published (90).

Another recommended rating scale (91) is the Scales for Outcomes in Parkinson's Disease (SCOPA)-Motor (27) for assessing motor functioning and disability in PD. It is composed by 21 items, scored from 0 (normal) to 3 (severe), and grouped into three sections: motor examination (10 items), activities of daily living (7 items), and motor complications (4 items). It does not include sleep problems. Satisfactory internal consistency, inter-rater and test-retest reliability and construct validity have been reported (92). It is also responsive to changes over time (93) and can predict an increase in PD-related costs (94).

The SCOPA-Autonomic (28), the first validated rating scale specifically designed for assessing autonomic symptoms in PD, includes two items on symptoms that can cause sleep disturbances: nicturia and excessive sweating during the night. This scale, composed by 25 items grouped in 6 domains (cardiovascular, gastrointestinal, urinary, thermoregulatory, pupillomotor, and sexual), meets criteria for “recommended” (95, 96).

A wide set of other rating scales for assessing specific symptoms and manifestations of PD are recommended by the IPMDS (9), but they are out of the scope of this review.

### Progressive supranuclear palsy (PSP)

Insomnia and impaired sleep architecture are the most common sleep abnormalities in PSP, and are more frequently described in PSP than in other atypical parkinsonisms (4). In particular, PSP patients can show a shorter total sleep time, a lower sleep efficiency and a lower percentage of REM sleep than controls (97).

The Progressive Supranuclear Palsy Rating Scale (PSPRS) (29) is the IPMDS Task Force recommended rating scale for assessing symptoms and associated disability of the PSP (98). It has 28 items, scored on a 3- or 5-point scale, and grouped into six dimensions: history (with an item on sleep difficulty), mental, bulbar, supranuclear ocular motor, and limb and gait/mildline examinations. The item named “Sleep difficulty” focused on insomnia and rated from 0 to 4 is included in the “History/Daily activities” section. The total scale score ranges from 0 to 100. The scale shows good inter-rater reliability and satisfactory predictive validity in relation to survival. The minimal clinically important worsening has been established in 5.7 points (99).

### Tics and tourette syndrome (TS)

Tics and TS can be associated with sleep disturbances such as insomnia and abnormal behaviors during sleep (100). Specific sleep architecture abnormalities, such as shorter REM latency and increased percentage of REM sleep, have also been reported in patients with TS (6). However, none of the IPMDS-recommended scales for tics and TS assesses sleep problems.

Five rating scales have been recommended for the IPMDS Task Force for assessment of tics and Tourette syndrome (101). The most widely used rating scale for motor and phonic tics is the Yale Global Tic Severity Scale (YGTSS) (30). It is a complex, rater-based tool, composed by items rating number, frequency, intensity, complexity, and interference of symptoms in a scoring scale from 0 (none/absent) to 5 (severe/always). The YGTSS yields total motor and phonic scores, an overall impairment rating and a global severity score. Its psychometric properties are satisfactory and thresholds of score changes due to clinical treatment are available (102, 103).

The Shapiro Tourette Syndrome Severity Scale (STSSS) (31) rates intensity of symptoms and interference with functioning, and it is reliable, valid, brief, and easy to administer. The Tourette Syndrome Clinical Global Impressions scale (TS-CGI) (32), also a brief scale, scores the overall adverse impact of tics. These two scales are less comprehensive than the YGTSS, as they do not include some aspects such as frequency, complexity, and distribution of tics. The Tourette's Disorder Scale (TODS) (33) rates overall tics severity but also assess comorbid behavioral symptoms: inattention, hyperactivity, obsessions, compulsions, aggression, and emotional symptoms. It shows excellent internal consistency and excellent inter-rater agreement and convergent and divergent validity (104).

Finally, the Premonitory Urge for Tic Disorders Scale (PUTS), the only specific scale for tic-related premonitory urges, presented satisfactory psychometric properties only for patients older than 10 years (34).

## Discussion

Several conditions, toxic agents, and metabolic dysfunctions can produce effects on brain structures and functional circuits in such a way that movement disorders and sleep disorders are manifested simultaneously. On the other hand, some movement disorders are associated with disturbed physiological sleep patterns, adding to the distress, and quality of life deterioration these patients suffer. Correct management of both types of disorders is mandatory and thus requires close evaluation and monitoring. However, as frequently occurs in the realm of movement disorders, the existence of non-motor symptoms and, specifically, sleep disturbances may remain undeclared (89) and underdiagnosed, missing the opportunity of appropriate treatment to improve the patient's health state.

Objective methods, based on wearable devices and technological developments, can be used for the appraisal of severity of movement disorders, sleep disturbances, and both types of conditions simultaneously when they are present in combination. Objective methods are also used for the diagnosis of several sleep disorders according to sleep disorder diagnostic criteria (periodic limb movement, obstructive sleep apneas, etc.), and particularly when the sleep dysfunction is due to several different causes as in atypical Parkinsonism or HD.

Polysomnography is very useful for this objective, but due to the complexity and costs of the sleep laboratories, this resource can be applied usually to a limited proportion of patients (105). Inertial sensors for capturing and recording movement during sleep are increasingly used and this is a rapidly growing field because progress in technology continuously offers easier to manage and portable devices that provide great amounts of information. The great advantage of these objective methods is that they furnish genuine measures providing real numbers based on physical phenomena.

The main disadvantages of rating scales are the influence of subjectivity in score assignment and the ordinal level of measurement (representing an ordered classification rather than real numerical values) adopted for the huge majority of them. Nonetheless, rating scales have the advantage of low cost, simplicity of application without need of special circumstances and settings, long time frame evaluation, and the multitude of facets they can assess. These characteristics have favored the wide use of these instruments in clinical daily practice and research.

Many of the rating scales described in this review are focused on the specific abnormal movement they evaluate and, therefore, do not include other elements for sleep assessment. However, the fact that several comprehensive scales that were designed to gather those components representing the most relevant aspects of the corresponding disorder, do not include sleep evaluations even when sleep disorders are frequently present in such condition, is striking. In general, it seems that the field of motor disorders has difficulty recognizing the presence of non-motor manifestations as disturbances causing important health problems to patients. The existence of similar non-motor symptoms in the general population (e.g., insomnia), although usually with lower prevalence and severity, may explain why these symptoms remained hidden to the attention of clinicians interested in movement disorders. In turn, the lack of systematic screening of these non-motor problems, has possibly led to the infradiagnosis and treatment of these disorders.

## Conclusions

The present review offers a rapid and pragmatic vision of the properties of the most used rating scales in those movement disorders with related sleep disorders and reflects how most scales do not cover the simultaneous evaluation of sleep disturbances. The inclusion of instruments for screening and appraisal of sleep disorders in the assessment of patients with, for example, essential tremor or chronic tics, and the development of new rating scales including items and domains for evaluation of sleep disorders in movement disorders will help to recognize the magnitude of the problem this combination represents.

## Author contributions

CR-B and MF conception and design of the study and wrote the first draft of the manuscript. MK, RB, and PM-M revised the work critically for important intellectual content. All authors contributed to manuscript revision, read, and approved the submitted version.

### Conflict of interest statement

The authors declare that the research was conducted in the absence of any commercial or financial relationships that could be construed as a potential conflict of interest.

## References

[1] Hobart JC Cano SJ Zajicek JP Thompson AJ. Rating scales as outcome measures for clinical trials in neurology: problems, solutions, and recommendations. Lancet Neurol. (2007) 6:1094–105. 10.1016/S1474-4422(07)70290-9 18031706

[2] Giannoccaro MP Antelmi E Plazzi G. Sleep and movement disorders. Curr Opin Neurol. (2013) 26:428–34. 10.1097/WCO.0b013e3283632cef 23787770

[3] Abbott SM Videnovic A. Sleep disorders in atypical Parkinsonism. Mov Disord Clin Pract. (2014) 1:89–96. 10.1002/mdc3.12025 24955381 PMC4061748

[4] Iranzo A Molinuevo JL Santamaría J Serradell M Martí MJ Valldeoriola F. Rapid-eye-movement sleep behaviour disorder as an early marker for a neurodegenerative disorder: a descriptive study. Lancet Neurol. (2006) 5:572–7. 10.1016/S1474-4422(06)70476-8 16781987

[5] Martino D Ganos C Pringsheim TM. Tourette syndrome and chronic tic disorders: the clinical spectrum beyond tics. Int Rev Neurobiol. (2017) 134:1461–90. 10.1016/bs.irn.2017.05.006 28805580

[6] Zoccolella S Savarese M Lamberti P Manni R Pacchetti C Logroscino G. Sleep disorders and the natural history of Parkinson's disease: the contribution of epidemiological studies. Sleep Med Rev. (2011) 15:41–50. 10.1016/j.smrv.2010.02.004 20634113

[7] Wu Y Wang X Wang C Sun Q Song N Zhou Y. Prevalence and clinical features of non-motor symptoms of essential tremor in Shanghai rural area. Parkinsonism Relat Disord. (2016) 22:15–20. 10.1016/j.parkreldis.2015.10.617 26777409

[8] Kurtis MM Balestrino R Rodriguez-Blazquez C Forjaz MJ Martinez-Martin P A review of scales to evaluate sleep disturbances in movement disorders. Front Neurol. (2018) 9:369 10.3389/fneur.2018.00369 29896152 PMC5986889

[9] Movement Disorders Society MDS Rating Scales [Internet]. Available online at: http://www.movementdisorders.org/MDS/Education/Rating-Scales.htm (Cited November 10, 2017).

[10] Barnes TR. A rating scale for drug-induced akathisia. Br J Psychiatry. (1989) 154:672–6. 2574607 10.1192/bjp.154.5.672

[11] Barnes TRE. The barnes akathisia rating scale–revisited. J Psychopharmacol. (2003) 17:365–70. 10.1177/0269881103174013 14870947

[12] Teixeira AL Maia DP Cardoso F. UFMG Sydenham's chorea rating scale (USCRS): reliability and consistency. Mov Disord. (2005) 20:585–91. 10.1002/mds.20377 15648075

[13] Unified Huntington's disease rating scale: reliability and consistency Mov Disord. (1996) 11:136–42. 10.1002/mds.870110204 8684382

[14] Comella CL Leurgans S Wuu J Stebbins GT Chmura T Dystonia study group. rating scales for dystonia: a multicenter assessment. Mov Disord. (2003) 18:303–12. 10.1002/mds.10377 12621634

[15] Burke RE Fahn S Marsden CD Bressman SB Moskowitz C Friedman J. Validity and reliability of a rating scale for the primary torsion dystonias. Neurology (1985) 35:73–7. 3966004 10.1212/wnl.35.1.73

[16] Consky E Lang A Clinical assessments of patients with cervical dystonia. In: Jankovic J Hallett M editors. Therapy with Botulinum Toxin. New York, NY: Marcel Dekker (1994). p. 221–37.

[17] Fahn S Tolosa E Marín C Clinical rating scale for tremor. In: Jankovic J Tolosa E editors. Parkinson's Disease and Movement Disorders. Baltimore, MD: Williams and Wilkins (1993). p. 271–80.

[18] Stacy MA Elble RJ Ondo WG Wu S-C Hulihan J TRS study group. Assessment of interrater and intrarater reliability of the Fahn-Tolosa-Marin Tremor Rating Scale in essential tremor. Mov Disord. (2007) 22:833–8. 10.1002/mds.21412 17343274

[19] Bain PG Findley LJ Assessing Tremor Severity: A Clinical Handbook. London: Gordon-Smith (1993).

[20] Louis ED Ottman R Ford B Pullman S Martinez M Fahn S. The Washington heights-inwood genetic study of essential tremor: methodologic issues in essential-tremor research. Neuroepidemiology (1997) 16:124–33. 9159767 10.1159/000109681

[21] Elble R Comella C Fahn S Hallett M Jankovic J Juncos JL. Reliability of a new scale for essential tremor. Mov Disord. (2012) 27:1567–9. 10.1002/mds.25162 23032792 PMC4157921

[22] Wenning GK Tison F Seppi K Sampaio C Diem A Yekhlef F. Development and validation of the Unified Multiple System Atrophy Rating Scale (UMSARS). Mov Disord. (2004) 19:1391–402. 10.1002/mds.20255 15452868

[23] Frucht SJ Leurgans SE Hallett M Fahn S. The unified myoclonus rating scale. Adv Neurol. (2002) 89:361–76. 11968461

[24] Goetz CG Fahn S Martinez-Martin P Poewe W Sampaio C Stebbins GT. Movement Disorder Society-sponsored revision of the Unified Parkinson's Disease Rating Scale (MDS-UPDRS): process, format, and clinimetric testing plan. Mov Disord. (2007) 22:41–7. 10.1002/mds.21198 17115387

[25] Chaudhuri KR Martinez-Martin P Brown RG Sethi K Stocchi F Odin P. The metric properties of a novel non-motor symptoms scale for Parkinson's disease: results from an international pilot study. Mov Disord. (2007) 22:1901–11. 10.1002/mds.21596 17674410

[26] Chaudhuri KR Martinez-Martin P Schapira AHV Stocchi F Sethi K Odin P. International multicenter pilot study of the first comprehensive self-completed nonmotor symptoms questionnaire for Parkinson's disease: the NMSQuest study. Mov Disord. (2006) 21:916–23. 10.1002/mds.20844 16547944

[27] Marinus J Visser M Stiggelbout AM Rabey JM Martínez-Martín P Bonuccelli U. A short scale for the assessment of motor impairments and disabilities in Parkinson's disease: the SPES/SCOPA. J Neurol Neurosurg Psychiatry (2004) 75:388–95. 10.1136/jnnp.2003.017509 14966153 PMC1738938

[28] Visser M Marinus J Stiggelbout AM Van Hilten JJ. Assessment of autonomic dysfunction in Parkinson's disease: the SCOPA-AUT. Mov Disord. (2004) 19:1306–12. 10.1002/mds.20153 15390007

[29] Golbe LI Ohman-Strickland PA. A clinical rating scale for progressive supranuclear palsy. Brain (2007) 130:1552–65. 10.1093/brain/awm032 17405767

[30] Leckman JF Riddle MA Hardin MT Ort SI Swartz KL Stevenson J. The yale global tic severity scale: initial testing of a clinician-rated scale of tic severity. J Am Acad Child Adolesc Psychiatry. (1989) 28:566–73. 2768151 10.1097/00004583-198907000-00015

[31] Shapiro AK Shapiro E. Controlled study of pimozide vs. placebo in Tourette's syndrome. J Am Acad Child Psychiatry (1984) 23:161–73. 6371107 10.1097/00004583-198403000-00007

[32] Leckman JF Towbin KE Ort SI Cohen DJ Clinical assessment of tic disorders severity. In: Cohen DJ Bruun RD Leckman JF editors. Tourette's Syndrome and Tic Disorders: Clinical Understanding and Treatment. New York, NY: John Wiley & Sons (1988).

[33] Shytle RD Silver AA Sheehan KH Wilkinson BJ Newman M Sanberg PR. The Tourette's Disorder Scale (TODS): development, reliability, and validity. Assessment (2003) 10:273–87. 10.1177/1073191103255497 14503651

[34] Woods DW Piacentini J Himle MB Chang S. Premonitory Urge for Tics Scale (PUTS): initial psychometric results and examination of the premonitory urge phenomenon in youths with Tic disorders. J Dev Behav Pediatr. (2005) 26:397–403. 16344654 10.1097/00004703-200512000-00001

[35] Walters AS Hening W Rubinstein M Chokroverty S. A clinical and polysomnographic comparison of neuroleptic-induced akathisia and the idiopathic restless legs syndrome. Sleep (1991) 14:339–45. 1682986

[36] Moser AD Epping E Espe-Pfeifer P Martin E Zhorne L Mathews K. A survey-based study identifies common but unrecognized symptoms in a large series of juvenile Huntington's disease. Neurodegener Dis Manag. (2017) 7:307–15. 10.2217/nmt-2017-0019 29043929

[37] Piano C Losurdo A Della Marca G Solito M Calandra-Buonaura G Provini F. Polysomnographic findings and clinical correlates in huntington disease: a cross-sectional cohort study. Sleep (2015) 38:1489–95. 10.5665/sleep.4996 25845698 PMC4531417

[38] Maskevich S Jumabhoy R Dao PDM Stout JC Drummond SPA. Pilot validation of ambulatory activity monitors for sleep measurement in Huntington's disease gene carriers. J Huntingt Dis. (2017) 6:249–53. 10.3233/JHD-170251 28968241

[39] Bellosta Diago E Pérez Pérez J Santos Lasaosa S Viloria Alebesque A Martínez Horta S Kulisevsky J. Circadian rhythm and autonomic dysfunction in presymptomatic and early Huntington's disease. Parkinsonism Relat Disord. (2017) 44:95–100. 10.1016/j.parkreldis.2017.09.013 28935191

[40] Baker CR Domínguez D JF Stout JC Gabery S Churchyard A Chua P. Subjective sleep problems in Huntington's disease: a pilot investigation of the relationship to brain structure, neurocognitive, and neuropsychiatric function. J Neurol Sci. (2016) 364:148–53. 10.1016/j.jns.2016.03.021 27084236

[41] Ben-Pazi H Stoner JA Cunningham MW. Dopamine receptor autoantibodies correlate with symptoms in Sydenham's chorea. PLoS ONE (2013) 8:e73516. 10.1371/journal.pone.0073516 24073196 PMC3779221

[42] Fusco C Ucchino V Frattini D Pisani F Della Giustina E. Acute and chronic corticosteroid treatment of ten patients with paralytic form of Sydenham's chorea. Eur J Paediatr Neurol. (2012) 16:373–8. 10.1016/j.ejpn.2011.12.005 22197452

[43] Barreto LB Maciel ROH Maia DP Teixeira AL Cardoso F. Parkinsonian signs and symptoms in adults with a history of Sydenham's chorea. Parkinsonism Relat Disord. (2012) 18:595–7. 10.1016/j.parkreldis.2011.11.002 22104009

[44] Teixeira AL Maia DP Cardoso F. The initial testing and the discrimination property of the UFMG Sydenham's Chorea Rating Scale (USCRS). Arq Neuropsiquiatr. (2005) 63:825–7. 10.1590/S0004-282X2005000500019 16258663

[45] Siesling S Zwinderman AH van Vugt JP Kieburtz K Roos RA. A shortened version of the motor section of the Unified Huntington's disease rating scale. Mov Disord. (1997) 12:229–34. 10.1002/mds.870120214 9087982

[46] Mestre TA van Duijn E Davis AM Bachoud-Lévi A-C Busse M Anderson KE. Rating scales for behavioral symptoms in Huntington's disease: critique and recommendations. Mov Disord. (2016) 31:1466–78. 10.1002/mds.26675 27296904

[47] Youssov K Dolbeau G Maison P Boissé M-F Cleret De Langavant L Roos R Unified Huntington's disease rating scale for advanced patients: validation and follow-up study. Mov Disord. (2013) 28:1717–23. 10.1002/mds.25654 24166899 PMC5071381

[48] Torres JAKL Rosales RL. Nonmotor symptoms in dystonia. Int Rev Neurobiol. (2017) 134:1335–71. 10.1016/bs.irn.2017.05.003 28805575

[49] Kuyper DJ Parra V Aerts S Okun MS Kluger BM The non-motor manifestations of dystonia: a systematic review. Mov Disord. (2011) 26:1206–17. 10.1002/mds.23709 21484874 PMC3652664

[50] Trotti LM Esper CD Feustel PJ Bliwise DL Factor SA. Excessive daytime sleepiness in cervical dystonia. Parkinsonism Relat Disord. (2009) 15:784–6. 10.1016/j.parkreldis.2009.04.007 19447065

[51] Sforza E Montagna P Defazio G Lugaresi E. Sleep and cranial dystonia. Electroencephalogr Clin Neurophysiol. (1991) 79:166–9. 1714808 10.1016/0013-4694(91)90135-q

[52] Albanese A Sorbo FD Comella C Jinnah HA Mink JW Post B. Dystonia rating scales: critique and recommendations. Mov Disord. (2013) 28:874–83. 10.1002/mds.25579 23893443 PMC4207366

[53] Rohl B Collins K Morgan S Cosentino S Huey ED Louis ED. Daytime sleepiness and nighttime sleep quality across the full spectrum of cognitive presentations in essential tremor. J Neurol Sci. (2016) 371:24–31. 10.1016/j.jns.2016.10.006 27871441 PMC5467974

[54] Chunling W Zheng X. Review on clinical update of essential tremor. Neurol Sci. (2016) 37:495–502. 10.1007/s10072-015-2380-1 26749268

[55] Louis ED. Non-motor symptoms in essential tremor: a review of the current data and state of the field. Parkinsonism Relat Disord. (2016) 22:S115–118. 10.1016/j.parkreldis.2015.08.034 26343494 PMC4764070

[56] Sengul Y Sengul HS Yucekaya SK Yucel S Bakim B Pazarci NK. Cognitive functions, fatigue, depression, anxiety, and sleep disturbances: assessment of nonmotor features in young patients with essential tremor. Acta Neurol Belg. (2015) 115:281–7. 10.1007/s13760-014-0396-6 25471376

[57] Lenka A Benito-León J Louis ED. Is there a Premotor Phase of Essential Tremor? Tremor Other Hyperkinetic Mov. (2017) 7:498. 10.7916/D80S01VK 29051842 PMC5633681

[58] Elble R Bain P Forjaz MJ Haubenberger D Testa C Goetz CG. Task force report: scales for screening and evaluating tremor: critique and recommendations. Mov Disord. (2013) 28:1793–800. 10.1002/mds.25648 24038576

[59] Bain PG Findley LJ Atchison P Behari M Vidailhet M Gresty M. Assessing tremor severity. J Neurol Neurosurg Psychiatry (1993) 56:868–73. 8350102 10.1136/jnnp.56.8.868 PMC1015140

[60] Louis ED Barnes L Wendt KJ Ford B Sangiorgio M Tabbal S. A teaching videotape for the assessment of essential tremor. Mov Disord. (2001) 16:89–93. 11215599 10.1002/1531-8257(200101)16:1<89::aid-mds1001>3.0.co;2-l

[61] Louis ED Ford B Bismuth B. Reliability between two observers using a protocol for diagnosing essential tremor. Mov Disord. (1998) 13:287–93. 9539343 10.1002/mds.870130215

[62] Louis ED Agnew A Gillman A Gerbin M Viner AS. Estimating annual rate of decline: prospective, longitudinal data on arm tremor severity in two groups of essential tremor cases. J Neurol Neurosurg Psychiatry (2011) 82:761–5. 10.1136/jnnp.2010.229740 21436230 PMC3696191

[63] Frucht SJ Bordelon Y Houghton WH Reardan D. A pilot tolerability and efficacy trial of sodium oxybate in ethanol-responsive movement disorders. Mov Disord. (2005) 20:1330–7. 10.1002/mds.20605 15986420

[64] Voller B Lines E McCrossin G Artiles A Tinaz S Lungu C. Alcohol challenge and sensitivity to change of the essential tremor rating assessment scale. Mov Disord. (2014) 29:555–8. 10.1002/mds.25667 24123358 PMC4189108

[65] Chang WS Chung JC Kim JP Chang JW. Simultaneous thalamic and posterior subthalamic electrode insertion with single deep brain stimulation electrode for essential tremor. Neuromodulation Technol Neural Interface (2013) 16:236–43. 10.1111/j.1525-1403.2012.00503.x 22985104

[66] Mostile G Fekete R Giuffrida JP Yaltho T Davidson A Nicoletti A. Amplitude fluctuations in essential tremor. Parkinsonism Relat Disord. (2012) 18:859–63. 10.1016/j.parkreldis.2012.04.019 22591578

[67] Ohshima Y Nakayama H Matsuyama N Hokari S Sakagami T Sato T. Natural course and potential prognostic factors for sleep-disordered breathing in multiple system atrophy. Sleep Med. (2017) 34:13–7. 10.1016/j.sleep.2017.01.020 28522081

[68] Videnovic A. Management of sleep disorders in Parkinson's disease and multiple system atrophy. Mov Disord. (2017) 32:659–68. 10.1002/mds.26918 28116784 PMC5435534

[69] Palma J-A Norcliffe-Kaufmann L Kaufmann H. Diagnosis of multiple system atrophy. Auton Neurosci Basic Clin. (2017). 211:15–25. 10.1016/j.autneu.2017.10.007 29111419 PMC5869112

[70] Krismer F Seppi K Tison F Sampaio C Zangerl A Peralta C. The unified multiple system atrophy rating scale: intrarater reliability. Mov Disord. (2012) 27:1683–5. 10.1002/mds.25181 23114993 PMC6231538

[71] Guo XY Cao B Lei F Huang L Chen K Song W. Clinical and polysomnographic features of patients with multiple system atrophy in Southwest China. Sleep Breath (2013) 17:1301–7. 10.1007/s11325-013-0839-y 23563911

[72] Matsushima M Yabe I Takahashi I Hirotani M Kano T Horiuchi K. Validity and reliability of a pilot scale for assessment of multiple system atrophy symptoms. Cereb Ataxias (2017) 4:11. 10.1186/s40673-017-0067-5 28680652 PMC5496135

[73] Matsushima M Yabe I Oba K Sakushima K Mito Y Takei A. Comparison of Different symptom assessment scales for multiple system atrophy. Cerebellum (2016) 15:190–200. 10.1007/s12311-015-0686-4 26093615

[74] Caviness JN Brown P. Myoclonus: current concepts and recent advances. Lancet Neurol. (2004) 3:598–607. 10.1016/S1474-4422(04)00880-4 15380156

[75] Montagna P Provini F Vetrugno R. Propriospinal myoclonus at sleep onset. Neurophysiol Clin. (2006) 36(5–6):351–5. 10.1016/j.neucli.2006.12.004 17336781

[76] Salas Puig J Tuñón A Vidal JA Mateos V Guisasola LM Lahoz CH. [Janz's juvenile myoclonic epilepsy: a little-known frequent syndrome. A study of 85 patients]. Med Clin. (1994) 103:684–9. 7808074

[77] van Zijl JC Beudel M Elting J-WJ de Jong BM van der Naalt J van den Bergh WM. The inter-rater variability of clinical assessment in post-anoxic myoclonus. Tremor Other Hyperkinetic Mov. (2017) 7:470. 10.7916/D81R6XBV 28966876 PMC5618111

[78] Hainque E Vidailhet M Cozic N Charbonnier-Beaupel F Thobois S Tranchant C. A randomized, controlled, double-blind, crossover trial of zonisamide in myoclonus-dystonia. Neurology (2016) 86:1729–35. 10.1212/WNL.0000000000002631 27053715

[79] Schrempf W Brandt MD Storch A Reichmann H. Sleep disorders in Parkinson's disease. J Park Dis. (2014) 4:211–21. 10.3233/JPD-130301 24796235

[80] Fahn S Elton R UPDRS program members Unified Parkinson's disease rating scale. In: Fahn S Marsden C Goldstein M Calne D editors. Recent Developments in Parkinson's Disease. Florham Park, NJ: Macmillan Healthcare Information (1987), p. 153–163.

[81] Horváth K Aschermann Z Acs P Bosnyák E Deli G Pál E. Is the MDS-UPDRS a good screening tool for detecting sleep problems and daytime sleepiness in Parkinson's disease? Park Dis. (2014) 2014:806169. 10.1155/2014/806169 25506041 PMC4251816

[82] Poewe W Hauser RA Lang A ADAGIO Investigators. Effects of rasagiline on the progression of nonmotor scores of the MDS-UPDRS. Mov Disord. (2015) 30:589–92. 10.1002/mds.26124 25545629

[83] Jafari N Pahwa R Nazzaro JM Arnold PM Lyons KE. MDS-UPDRS to assess non-motor symptoms after STN DBS for Parkinson's disease. Int J Neurosci. (2016) 126:25–9. 10.3109/00207454.2015.1065257 26134878

[84] Horváth K Aschermann Z Ács P Deli G Janszky J Komoly S. Minimal clinically important difference on the Motor Examination part of MDS-UPDRS. Parkinsonism Relat Disord. (2015) 21:1421–6. 10.1016/j.parkreldis.2015 26578041

[85] Martinez-Martin P Rodriguez-Blazquez C Abe K Bhattacharyya KB Bloem BR Carod-Artal FJ. International study on the psychometric attributes of the non-motor symptoms scale in Parkinson disease. Neurology (2009) 73:1584–91. 10.1212/WNL.0b013e3181c0d416 19901251

[86] Chaudhuri KR Martinez-Martin P Antonini A Brown RG Friedman JH Onofrj M Rotigotine and specific non-motor symptoms of Parkinson's disease: *post hoc* analysis of RECOVER. Parkinsonism Relat Disord. (2013) 19:660–5. 10.1016/j.parkreldis.2013.02.018 23557594

[87] Martinez-Martin P Reddy P Katzenschlager R Antonini A Todorova A Odin P. EuroInf: a multicenter comparative observational study of apomorphine and levodopa infusion in Parkinson's disease. Mov Disord. (2015) 30:510–6. 10.1002/mds.26067 25382161

[88] Perez Lloret S Rossi M Cardinali DP Merello M. Validation of the sleep related items of the Non-motor Symptoms Questionnaire for Parkinson's disease (NMSQuest). Parkinsonism Relat Disord. (2008) 14:641–5. 10.1016/j.parkreldis.2008.01.004 18329317

[89] Chaudhuri KR Prieto-Jurcynska C Naidu Y Mitra T Frades-Payo B Tluk S. The nondeclaration of nonmotor symptoms of Parkinson's disease to health care professionals: an international study using the nonmotor symptoms questionnaire. Mov Disord. (2010) 25:704–9. 10.1002/mds.22868 20437539

[90] Chaudhuri KR Sauerbier A Rojo JM Sethi K Schapira AHV Brown RG. The burden of non-motor symptoms in Parkinson's disease using a self-completed non-motor questionnaire: a simple grading system. Parkinsonism Relat Disord. (2015) 21:287–91. 10.1016/j.parkreldis.2014.12.031 25616694

[91] Shulman LM Armstrong M Ellis T Gruber-Baldini A Horak F Nieuwboer A. Disability rating scales in Parkinson's disease: critique and recommendations. Mov Disord. (2016) 31:1455–65. 10.1002/mds.26649 27193358

[92] Martínez-Martín P Benito-León J Burguera JA Castro A Linazasoro G Martínez-Castrillo JC. The SCOPA-Motor Scale for assessment of Parkinson's disease is a consistent and valid measure. J Clin Epidemiol. (2005) 58:674–9. 10.1016/j.jclinepi.2004.09.014 15939218

[93] Marinus J van der Heeden JF van Hilten JJ. Calculating clinical progression rates in Parkinson's disease: methods matter. Parkinsonism Relat Disord. (2014) 20:1263–7. 10.1016/j.parkreldis.2014.08.009 25264021

[94] Martinez-Martín P Rodriguez-Blazquez C Paz S Forjaz MJ Frades-Payo B Cubo E. Parkinson symptoms and health related quality of life as predictors of costs: a longitudinal observational study with linear mixed model analysis. PLoS ONE (2015) 10:e0145310. 10.1371/journal.pone.0145310 26698860 PMC4689528

[95] Evatt ML Chaudhuri KR Chou KL Cubo E Hinson V Kompoliti K. Dysautonomia rating scales in Parkinson's disease: sialorrhea, dysphagia, and constipation–critique and recommendations by movement disorders task force on rating scales for Parkinson's disease. Mov Disord. (2009) 24:635–46. 10.1002/mds.22260 19205066 PMC4404514

[96] Pavy-Le Traon A Amarenco G Duerr S Kaufmann H Lahrmann H Shaftman SR. The Movement Disorders task force review of dysautonomia rating scales in Parkinson's disease with regard to symptoms of orthostatic hypotension. Mov Disord. (2011) 26:1985–992. 10.1002/mds.23742 21547951

[97] Montplaisir J Petit D Décary A Masson H Bédard MA Panisset M. Sleep and quantitative EEG in patients with progressive supranuclear palsy. Neurology (1997) 49:999–1003. 9339679 10.1212/wnl.49.4.999

[98] Hall DA Forjaz MJ Golbe LI Litvan I Payan CAM Goetz CG Scales to assess clinical features of progressive supranuclear palsy: MDS task force report. Mov Disord Clin Pract. (2015) 2:127–34. 10.1002/mdc3.12130 PMC6183172 30363842

[99] Hewer S Varley S Boxer AL Paul E Williams DR AL-108-231 Investigators. Minimal clinically important worsening on the progressive supranuclear Palsy Rating Scale. Mov Disord. (2016) 31:1574–7. 10.1002/mds.26694 27324431 PMC5215805

[100] Ghosh D Rajan PV Das D Datta P Rothner AD Erenberg G. Sleep disorders in children with Tourette syndrome. Pediatr Neurol. (2014) 51:31–5. 10.1016/j.pediatrneurol.2014.03.017 24938137

[101] Martino D Pringsheim TM Cavanna AE Colosimo C Hartmann A Leckman JF. Systematic review of severity scales and screening instruments for tics: critique and recommendations. Mov Disord. (2017) 32:467–73. 10.1002/mds.26891 28071825 PMC5482361

[102] Storch EA De Nadai AS Lewin AB McGuire JF Jones AM Mutch PJ. Defining treatment response in pediatric tic disorders: a signal detection analysis of the Yale global tic severity scale. J Child Adolesc Psychopharmacol. (2011) 21:621–7. 10.1089/cap.2010.0149 22070181 PMC3279714

[103] Jeon S Walkup JT Woods DW Peterson A Piacentini J Wilhelm S. Detecting a clinically meaningful change in tic severity in Tourette syndrome: a comparison of three methods. Contemp Clin Trials (2013) 36:414–20. 10.1016/j.cct.2013.08.012 24001701 PMC3999642

[104] Storch EA Merlo LJ Lehmkuhl H Grabill KM Geffken GR Goodman WK. Further psychometric examination of the Tourette's Disorder Scales. Child Psychiatry Hum Dev. (2007) 38:89–98. 10.1007/s10578-006-0043-4 17136450

[105] Bhidayasiri R Martinez-Martin P. Clinical Assessments in Parkinson's disease: scales and monitoring. Int Rev Neurobiol. (2017) 132:129–82. 10.1016/bs.irn.2017.01.001 28554406

